# Quantitative ultrasound radiomics using texture derivatives in prediction of treatment response to neo-adjuvant chemotherapy for locally advanced breast cancer

**DOI:** 10.18632/oncotarget.27742

**Published:** 2020-10-20

**Authors:** Archya Dasgupta, Stephen Brade, Lakshmanan Sannachi, Karina Quiaoit, Kashuf Fatima, Daniel DiCenzo, Laurentius O. Osapoetra, Murtuza Saifuddin, Maureen Trudeau, Sonal Gandhi, Andrea Eisen, Frances Wright, Nicole Look-Hong, Ali Sadeghi-Naini, William T. Tran, Belinda Curpen, Gregory J. Czarnota

**Affiliations:** ^1^Department of Radiation Oncology, Sunnybrook Health Sciences Centre, Toronto, Canada; ^2^Department of Radiation Oncology, University of Toronto, Toronto, Canada; ^3^Physical Sciences, Sunnybrook Research Institute, Toronto, Canada; ^4^Department of Medical Oncology, Department of Medicine, Sunnybrook Health Sciences Centre, Toronto, Canada; ^5^Department of Medicine, University of Toronto, Toronto, Canada; ^6^Department of Surgical Oncology, Department of Surgery, Sunnybrook Health Sciences Centre, Toronto, Canada; ^7^Department of Surgery, University of Toronto, Toronto, Canada; ^8^Department of Medical Biophysics, University of Toronto, Toronto, Canada; ^9^Department of Electrical Engineering and Computer Sciences, Lassonde School of Engineering, York University, Toronto, Canada; ^10^Evaluative Clinical Sciences, Sunnybrook Research Institute, Toronto, Canada; ^11^Department of Medical Imaging, Sunnybrook Health Sciences Centre, Toronto, Canada; ^12^Department of Medical Imaging, University of Toronto, Toronto, Canada

**Keywords:** radiomics, breast cancer, texture derivatives, quantitative ultrasound, neoadjuvant chemotherapy

## Abstract

Background: To investigate quantitative ultrasound (QUS) based higher-order texture derivatives in predicting the response to neoadjuvant chemotherapy (NAC) in patients with locally advanced breast cancer (LABC).

Materials and Methods: 100 Patients with LABC were scanned before starting NAC. Five QUS parametric image-types were generated from radio-frequency data over the tumor volume. From each QUS parametric-image, 4 grey level co-occurrence matrix-based texture images were derived (20 QUS-Tex^1^), which were further processed to create texture derivatives (80 QUS-Tex^1^-Tex^2^). Patients were classified into responders and non-responders based on clinical/pathological responses to treatment. Three machine learning algorithms based on linear discriminant (FLD), *k*-nearest-neighbors (KNN), and support vector machine (SVM) were used for developing radiomic models of response prediction.

Results: A KNN-model provided the best results with sensitivity, specificity, accuracy, and area under curve (AUC) of 87%, 81%, 82%, and 0.86, respectively. The most helpful features in separating the two response groups were QUS-Tex^1^-Tex^2^ features. The 5-year recurrence-free survival (RFS) calculated for KNN predicted responders and non-responders using QUS-Tex^1^-Tex^2^ model were comparable to RFS for the actual response groups.

Conclusions: We report the first study demonstrating QUS texture-derivative methods in predicting NAC responses in LABC, which leads to better results compared to using texture features alone.

## INTRODUCTION

Breast cancer is the second most common cancer globally in terms of incidence, comprising 11.6% of all new cancers and is the 5th leading cause of mortality attributed to 6.6% of all cancer deaths [1]. For patients with locally advanced breast cancer (LABC), pre-operative or neoadjuvant systemic therapies with chemotherapy with or without targeted agents constitute standard treatment protocols [2–4]. The rationale of using NAC is to downsize the primary tumor improving resectability, and also the assessment of response to therapies that serve as a reliable prognostic marker of outcomes. Imaging modalities like ultrasonography (USG), mammography (MMG), magnetic resonance imaging (MRI), and computed tomography (CT) are commonly used in response monitoring of NAC, which primarily considers long-term size related changes of the disease [5, 6]. Also, functional or metabolic imaging in the form of positron emission tomography (PET) is recognized in evaluating treatment response.

Radiomics is an emerging field in medicine and oncology, which has been made possible through advanced computer-based image analysis, often combined with machine learning algorithms for data interpretation [7–9]. Radiomics has made it possible to extract biological characteristics in a non-invasive manner, which can help in predicting the natural history of the disease as well as in detecting early changes associated with treatment. Quantitative ultrasound (QUS) is a non-invasive, easily accessible, and relatively inexpensive imaging modality that can lead to useful insights into the tissue microstructure [10]. Radiofrequency (RF) data from QUS provides valuable information compared to conventional B-mode ultrasound imaging, where there is a loss of crucial details involved with instrument-based signal processing. The analysis of the power spectra from ultrasound RF data can be used to determine quantitative parameters, which include average scatterer diameter (ASD), average acoustic concentration (AAC), mid-band fit (MBF), spectral slope (SS), spectral 0-MHz intercept (SI). These reveal useful information about elastic properties in tissue at the microscopic level, with each parameter having distinct biological implications [11]. Also, QUS features are capable of detecting temporal events at the cellular level following various treatments, including cell death [12, 13]. The spatial distribution of features within QUS parametric images can be further studied using grey-level co-occurrence matrix (GLCM) analyses, which represent the angular relationship and distance between neighboring pixels. The GLCM method can be used to extract various QUS-texture (QUS-Tex^1^) features like contrast (CON), energy (ENE), correlation (COR), and homogeneity (HOM). Earlier studies have evaluated baseline QUS parameters and texture analysis extracted from the tumor to predict the response to systemic therapies [14, 15].

In the study here, higher-order imaging features in the form of QUS texture-derivatives (QUS-Tex^1^-Tex^2^) have been determined from pretreatment QUS data for patients with LABC undergoing NAC to predict treatment response. To the best of our knowledge, this is the first study to demonstrate the effectiveness of texture derivatives applied to any form of imaging data, leading to the improvement of classifier performances.

## RESULTS

### Patient, tumor and treatment characteristics

Patient and treatment-related features are summarized in Table 1. According to the clinical and pathological criteria, 83 and 17 patients were classified as responders and non-responders to NAC, respectively. The most common chemotherapy regimen used was AC-T (Adriamycin, Cyclophosphamide, and Paclitaxel) in 59%, followed by FEC-D (5-Fluorouracil, Epirubicin, Cyclophosphamide, and Docetaxel) in 29%. Along with neoadjuvant chemotherapy, 31% of patients received trastuzumab in the neoadjuvant setting. None of the patients received any endocrine therapy before surgery. All of the patients included in this analysis had completed systemic therapy as planned. Pathological complete response (pCR) was seen in 23 patients (23%). Following surgery adjuvant therapies with radiation, maintenance Trastuzumab for human epidermal growth factor receptor 2 (HER2) positive tumors or endocrine therapy (for hormonal receptor-positive) was continued as per standard institutional practice. Details on individual patient basis have been mentioned in Supplementary Table 1. The 5-year recurrence-free survival (RFS) for the responders and non-responders was 84% and 62%, respectively, with a *p*-value of 0.04 (Supplementary Figure 1).

**Table 1:** Patient, disease and treatment related characteristics in the two groups (responders and non-responders)

Characteristics	Responders (*n* = 83)	Non-responders (*n* = 17)	Total (*n* = 100)	*p*-value
**Age**				
Median (range)	50 (31–84) years	42 (29–66) years	49 (29–84) years	0.25
**Menopausal status**				
Premenopausal	46 (55%)	11 (65%)	57 (57%)	
Postmenopausal	29 (35%)	5 (30%)	34 (34%)	0.85
Perimenopausal	5 (6%)	0 (0%)	5 (5%)	
Unknown	3 (4%)	1 (5%)	4 (4%)	
**Tumour size (baseline)**				
Median (range)	5.3 (1.6–12) cm	5.3 (2.5–12.8) cm	5.3 (1.6–12.8) cm	0.62
**Histology**				
IDC	76 (92%)	14 (82%)	90 (90%)	
ILC	4 (5%)	1 (6%)	5 (5%)	0.36
Others	3 (3%)	2 (12%)	5 (5%)	
**Tumour stage**				
T1	0 (0%)	0 (0%)	0 (0%)	
T2	34 (41%)	8 (47%)	42 (42%)	0.89
T3	39 (47%)	7 (41%)	46 (46%)	
T4	10 (12%)	2 (12%)	12 (12%)	
**Nodal stage**				
N0	21 (25%)	5 (29%)	26 (26%)	
N1	51 (61%)	8 (47%)	59 (59%)	0.14
N2	8 (10%)	1 (6%)	9 (9%)	
N3	3 (4%)	3 (18%)	6 (6%)	
**Hormonal status**				
Positive	51 (61%)	12 (71%)	63 (63%)	0.48
Negative	32 (39%)	5 (29%)	37 (37%)	
**HER2 status**				
Positive	28 (34%)	3 (18%)	31 (31%)	0.19
Negative	55 (66%)	14 (82%)	69 (69%)	
**Systemic therapy**				
AC-T	51 (61%)	8 (47%)	59 (59%)	
FEC-D	22 (27%)	7 (41%)	29 (29%)	0.46
Others	10 (12%)	2 (12%)	12 (12%)	
**Trastuzumab**				
Yes	28 (34%)	3 (18%)	31 (31%)	0.19
No	55 (66%)	14 (82%)	69 (69%)	
**Surgery**				
Mastectomy	64 (77%)	15 (88%)	79 (79%)	
Breast-conserving surgery	19 (23%)	1 (6%)	20 (20%)	0.13
No surgery	0 (0%)	1 (6%)	1 (1%)	
**Tumour size (post-NAC)**				
Median (range)	1.4 (0–8.4) cm	5 (2.4–19.0) cm	1.8 (0–19.0) cm	< 0.01

Abbreviations: IDC: Infiltrating duct carcinoma; ILC: Infiltrating lobular carcinoma; HER2: Human epidermal growth factor receptor 2; AC-T: Adriamycin, Cyclophosphamide, and Paclitaxel; FEC-D: 5-Fluorouracil, Epirubicin, Cyclophosphamide, and Docetaxel; NAC: Neoadjuvant chemotherapy.

### Quantitative ultrasound and texture parameters

**Figure 1:**
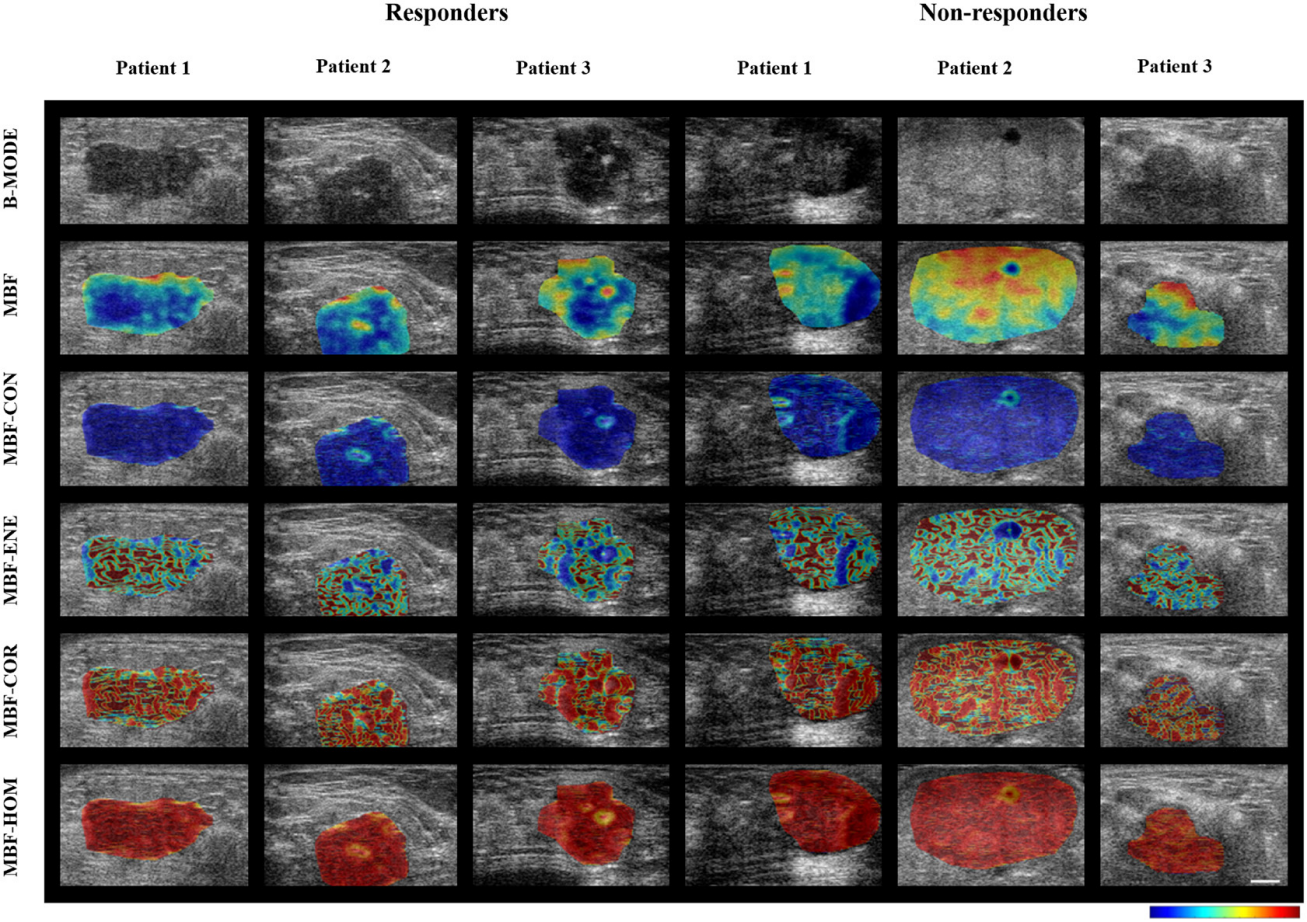
Parametric maps for the two response groups. Representative ultrasound B-mode images with MBF parameter overlays, and MBF-CON, MBF-ENE, MBF-COR, and MBF-HOM feature overlays acquired from three responder and non-responder patients before chemotherapy. To generate the parametric maps, the values were normalized across all the patients (irrespective of treatment response) for individual features. The normalized values for the feature were then represented quantitatively as colors across the sub-regions of interest across the entire tumor volume. The scale bar in ultrasound images represents 5 mm. The color bars represent scales for the MBF parameter of -9 to 18 dBr, for the MBF-CON parameter of 0 to 2.7, for MBF-ENE parameter of 0 to 1, for the MBF-COR parameter of -0.4 to 0.9, and for MBF-HOM parameter of 0 to 1. MBF: Mid-band fit; CON: Contrast; ENE: Energy; COR: Correlation; HOM: Homogeneity.

**Figure 2:**
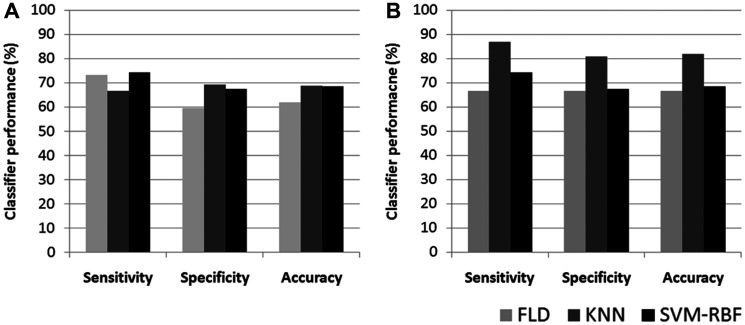
Bar diagram of classifier performance for different models. Classification performance represented as bar diagram based on QUS + QUS-Tex^1^ (**A**) and QUS + QUS-Tex^1^ + QUS-Tex^1^-Tex^2^ parameters (**B**) using FLD, KNN, and SVM-RBF classifiers. QUS: Quantitative ultrasound; FLD: Fisher’s linear discriminant; SVM: Support vector machine; KNN: k-nearest-neighbors.

**Figure 3:**
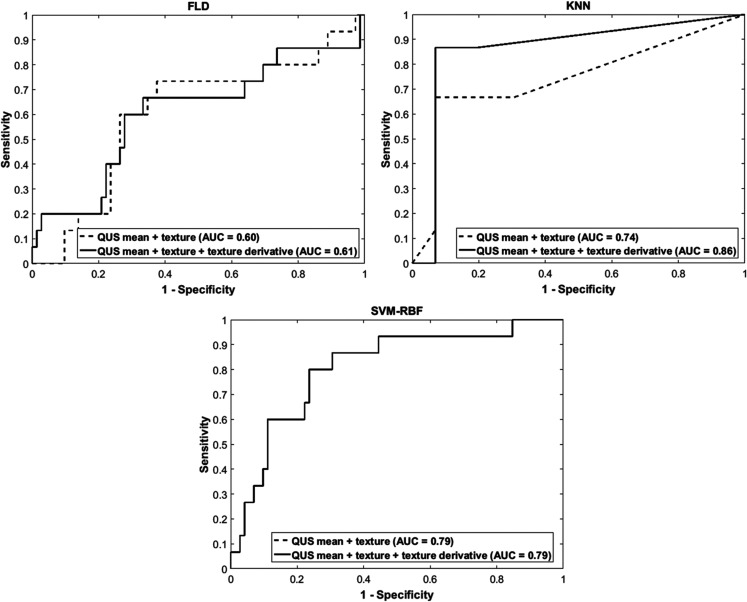
Receiver operator characteristics curves for different models. Receiver operator characteristics curves for the response predictor model obtained based on QUS+QUS-Tex^1^ (dotted line) and based on QUS + QUS-Tex^1^ + QUS-Tex^1^-Tex^2^ (solid line) using FLD, SVM, and KNN classifiers. QUS: Quantitative ultrasound; FLD: Fisher’s linear discriminant; SVM: Support vector machine; KNN: k-nearest-neighbors; AUC: Area under curve.

Representative ultrasound B-mode images, MBF parametric images and MBF-CON, MBF-ENE, MBF-COR, and MBF-HOM texture images corresponding to three responding and three non-responding patients are displayed in Figure 1. None of the parameters demonstrated significant differences in terms of distribution between the two groups. The classification performances using the different algorithms have been summarized in Table 2. The accuracies from QUS and QUS-Tex^1^ features using Fisher’s linear discriminant (FLD), k-nearest neighbors (KNN), and radial-basis-function support vector machine (SVM) classifiers were 62%, 69%, and 69%, respectively. Selecting from 105 features inclusive of the texture derivatives, accuracy improved to 67%, 82%, and 69% for FLD, KNN, and SVM, respectively. Amongst all the models, KNN classification resulted in the best performances. The sensitivity, specificity, accuracy, and area under curve (AUC) for the best classifier was 87%, 81%, 82%, and 0.86, respectively. The comparison of the performance using QUS+QUS-Tex^1^ and QUS-Tex^1^-Tex^2^ is shown in Figure 2A and 2B, respectively. The Receiver operating characteristics (ROC) curves (Figure 3) indicate the improvement in AUC values for the KNN model (0.86 vs. 0.74), whereas it remained relatively stable for FLD (0.61 vs. 0.60) and SVM (0.79 both) methods.

**Table 2:** Classifier performance for different machine learning models using QUS-Tex^1^ and QUS-Tex^1^-Tex^2^

Classifier	Features	Sensitivity	Specificity	PPV	NPV	Accuracy	AUC	Selected parameters
**FLD**	**QUS-Tex^1^**	73%	60%	28%	92%	62%	0.60	SI-COR
	**QUS-Tex^1^-Tex^2^**	67%	67%	29%	91%	67%	0.61	SI-COR
**KNN**	**QUS-Tex^1^**	67%	69%	31%	91%	69%	0.74	SI-COR
	**QUS-Tex^1^-Tex^2^**	**87%**	**81%**	**48%**	**97%**	**82%**	**0.86**	AAC-CON-ENE, MBF-COR-ENE, SI-COR-ENE
**SVM**	**QUS-Tex^1^**	74%	67%	33%	94%	69%	0.79	MBF, AAC-ENE, ASD-ENE
	**QUS-Tex^1^-Tex^2^**	74%	67%	33%	94%	69%	0.79	SI-ENE, ASD-ENE-CON, MBF-COR-COR

Abbreviations: QUS: Quantitative ultrasound; QUS-Tex^1^: QUS first-order texture; QUS-Tex^1^-Tex^2^: QUS-texture derivatives; FLD: Fisher’s linear discriminant; SVM: Support vector machine; KNN: k-nearest-neighbors; SI: Spectral 0-MHz intercept; MBF: Mid-band fit; AAC: Average acoustic concentration; ASD: Average scatterer diameter; SS: Spectral slope; COR: Correlation; ENE: Energy; CON: Contrast; PPV: Positive predictive value; NPV: Negative predictive value.

The best-selected features for model development are summarized in Table 2. The model used 1 feature for classification when no further improvement (or decrease) in the classifier performance was evident on the subsequent addition of 2nd or 3rd features (for FLD both models and KNN-texture model). The best classifier model (KNN-texture derivatives) used three higher-order features (AAC-CON-ENE, MBF-COR-ENE, SI-COR-ENE) for the group classification.

## DISCUSSION

Locally advanced breast cancer encompasses stage III breast cancer, denoting a higher disease burden, either with an extensive primary disease (T_3_, T_4_) or advanced nodal involvement (N_2_, N_3_) or both [16]. With existing treatment protocols, the five-year overall survival has been reported to be 50–80% and is influenced by clinical, molecular, and treatment-related features [16–19]. Often, due to the extensive nature of the disease, upfront surgery appears challenging for patients with LABC. Systemic therapies before surgery have been shown to result in downsizing tumors and improve resectability [4, 19–21]. Also, the use of NAC permits the assessment of response to systemic treatment, which may serve as a reliable prognostic marker for survival [22]. Response monitoring during therapy with various imaging modalities with the early identification of non-responders provides a window for adopting personalized medicine. Similarly, the anticipation of response before the initiation of treatment may further facilitate decision making by permitting appropriate therapies to be selected. In this study, a novel approach is presented using QUS higher-order texture derivatives in predicting the response to NAC.

Several strategies have been explored for response prediction and monitoring, including molecular analysis and different imaging modalities. It has been shown that the response rates to NAC are dependent on the molecular profiles, with pCR being higher for HER2 overexpression and triple-negative breast cancer subtypes [23, 24]. Molecular signatures based on stromal-related genes have been able to predict resistance to neoadjuvant chemotherapy [25]. Similarly, transcriptional profiling had been used to develop a multigene model to predict pCR in breast cancer patients treated with neoadjuvant paclitaxel and fluorouracil, doxorubicin, and cyclophosphamide [26]. Recently, the circulating tumor DNA (ctDNA) has been used as a potential biomarker for response monitoring in breast cancer receiving NAC [27, 28].

Imaging constitutes an integral role in the current oncological practice. Various imaging modalities like MMG, USG, CT, MRI, and PET-CT are used in the management of patients with breast cancer in diagnostic work up, staging, therapy guidance, response evaluation, and surveillance following treatment completion. Radiomics can help in determining imaging features that can lead to information reflecting underlying cellular biology predicting biological behavior. Literature is limited regarding the utility of baseline imaging features to predict response to NAC or survival outcomes in breast cancer. A study involving patients with triple-negative breast cancer (TNBC) analyzing clinic-pathological factors, found that the presence of microcalcifications on baseline imaging was associated with predicting residual disease as opposed to patients with pCR [29]. Radiogenomics involves the integration of imaging characteristics with molecular information and has shown promising results in breast cancer using baseline MRI features [30]. Texture analysis of T1-weighted dynamic contrast-enhanced MRI features has been used to predict response to NAC in a set of 58 patients with LABC [31]. In previous studies, QUS features from breast tumors were indicated to predict response to NAC and predict survival in a cohort of 56 patients with LABC [14]. The work here extended that data to include 100 patients and uses higher-order features to improve classifier performance further.

The clinical application of QUS spectral and texture analysis to identify a subset of patients likely to respond to NAC as early as four weeks (serial scans) or even before the initiation of treatment (baseline imaging) [14, 32, 33]. In the work here, we included five spectral features and generated a set of 20 texture (QUS-Tex^1^) and, finally, 80 higher-order texture derivatives (QUS-Tex^1^-Tex^2^). Each spectral parameter reflects different aspects of underlying cellular biology; MBF, ASD, and AAC can be related to microstructure and cellular organization, scatterer size, and scatterer number density.

In our previous work involving comparative analysis of QUS parameters from pretreatment and intra-treatment scans, the most evident changes were noticed in mean values of MBF and SI, suggesting microenvironment changes in response to NAC [32]. Here, the KNN model exhibited the best diagnostic matrices, with all the three features selected by the algorithm being texture-derivatives. This resulted in better classifier performance, as evident from improvements in AUC. It is possible that the second-order texture derivatives represent intratumoral heterogeneity better, therefore leading to improved prediction of clinical outcomes. In addition, it may be possible with further sequential texture analysis that underlying information is extracted at finer levels, which otherwise is obscured.

As expected, clinical outcomes were distinctly different between the responders and non-responders as defined by the clinical-pathological criteria and demonstrated in survival plots. This reflects the strong influence of response to neoadjuvant treatment on tumor biological behavior and outcomes depicted by a relapse of breast cancer. It is essential to note although the sensitivity of the three models was comparable (Figure 2B), the specificity and accuracy were higher for classification using the KNN model.

The early identification of patients not responding to NAC does have potentially significant clinical implications. Alternative chemotherapy regimens or upfront surgery should be considered in patients who are refractory to the standard protocols rather than continuing with the ineffective regimens. In a phase 2 randomized study involving HER2+ breast cancer, PET was used for response assessment before the 1st and 2nd cycles of neoadjuvant therapy with docetaxel and trastuzumab. Predicted non-responders were randomly assigned to 4 cycles of the same treatment or the addition of bevacizumab. The addition of bevacizumab for predicted non-responders resulted in better pathological complete response rates [34]. Other studies have indicated a benefit to intensifying treatment in non-responding patients [35]. In addition, there is recent interest in avoiding surgery for patients achieving pCR to NAC, particularly in patients with TNBC or HER2+ tumors, being explored in ongoing trials [36]. The QUS-based model has the potential of identifying such patients ahead of time, helping in treatment decisions.

Based on the results from previous observational studies, the clinical utility of QUS-radiomics is being evaluated in a randomized clinical trial, where patients predicted to have inadequate response can have modifications made to their NAC (http://ClinicalTrials.gov identifier NCT04050228). In that work, the goals are the identification of biological non-responders as soon as the first cycle of chemotherapy to modify the protocol early and avoid unnecessary treatments; the identification of pathological complete responders to possibly avoid surgical procedures.

QUS is a simple, easily accessible imaging modality, with similar scanning techniques akin to the B-mode US, which is widely used in clinical practice. With appropriate data processing, it is possible to use the information normally processed to generate B-mode images to obtain additional information from tumors. These are related to tumor acoustic properties, which can be linked with biological features and clinical behavior. In this research, we established the role of higher-order imaging analysis (radiomics) of QUS in predicting treatment response to NAC involving 100 patients with LABC. The work provides a framework for using QUS-radiomics in clinical practice to choose appropriate chemotherapy regimens or other treatment modalities like upfront surgery (in predicted chemo-resistant tumors), leading the way towards personalized oncology.

## MATERIALS AND METHODS

### Patient selection and treatment protocols

All the patients included in this report were obtained from a prospective study approved by the Sunnybrook Health Sciences Centre research ethics board and registered with http://ClinicalTrials.gov (NCT00437879). Patients accrued between January 2009 and January 2017 were included in the current analysis. Written informed consent was obtained from patients before accrual to study. Patients with LABC receiving NAC were considered eligible for the study. All patients were required to have a histological diagnosis of primary breast malignancy before initiation of any treatment with documentation of pathological and molecular information like hormonal receptor or HER 2 status. The choice regarding the NAC regimen and timing was at the discretion of the treating medical oncologist, with almost all patients receiving anthracycline and taxane-based chemotherapy regimens. Also, patients receiving HER 2 targeted therapies in addition to neoadjuvant chemotherapy were allowed in the study.

Following surgery, surgical specimens were evaluated by a board-certified pathologist for the assessment of histological characteristics and tumor response. Patients were classified into responders and non-responders based on the combined clinical and pathological evaluation. This modified response grading system was based on combined radiological and pathological assessment, and had been described in our previous studies [14, 15, 37]. Responders (R) were required to have tumor reduction by at least 30% of pretreatment dimensions, and/or a decrease in cellularity (residual invasive and *in-situ* < 1%), with all other patients being considered as non-responders (NR).

### Ultrasound data acquisition

As a part of this study, ultrasound RF data was acquired using a Sonix RP clinical research system (Analogic Medical Corp., Vancouver) with a linear array transducer (L14-5/60, Analogic Medical Corp., Vancouver) having a central frequency of 7 MHz (bandwidth 4–9 MHz). Imaging was conducted before the initiation of NAC. The sampling frequency of 40 MHz was considered for digital acquisition of ultrasound data with a 16-bit resolution. The tumor was scanned at intervals of 1 cm across the breast to cover the entire range of the primary tumor, with the transducer focused towards the center of the tumor. For the determination of QUS features, the region of interest (ROI) was manually drawn corresponding to the edge of the tumor in all image planes. A recent publication had shown the effects associated with different clinical ultrasound systems, or other factors related to image acquisition can be overcome with appropriate normalization techniques [38]. All the acquired images related to the selected slices for image extraction, segmentation of the tumor were verified by the principal investigator (GJC).

### Feature extraction

Quantitative ultrasound parametric maps, including MBF, SS, SI, ASD, and AAC, were determined using quantitative ultrasound techniques described in previous studies [11]. A reference phantom method was used to remove any clinical system dependencies in QUS parameters estimation and standardization. The entire ROI was divided into window blocks (considered as sub-ROIs) of size 10l × 10l with a 94% overlap in axial and lateral directions, where l represents the ultrasound wavelength. This corresponded to 15 by 15 pixels or 2.2 mm by 2.2 mm approximately. For each of the QUS features, values were determined from each sub-ROIs assigning specific values on a quantitative scale, leading to the generation of QUS parametric maps.

**Figure 4:**
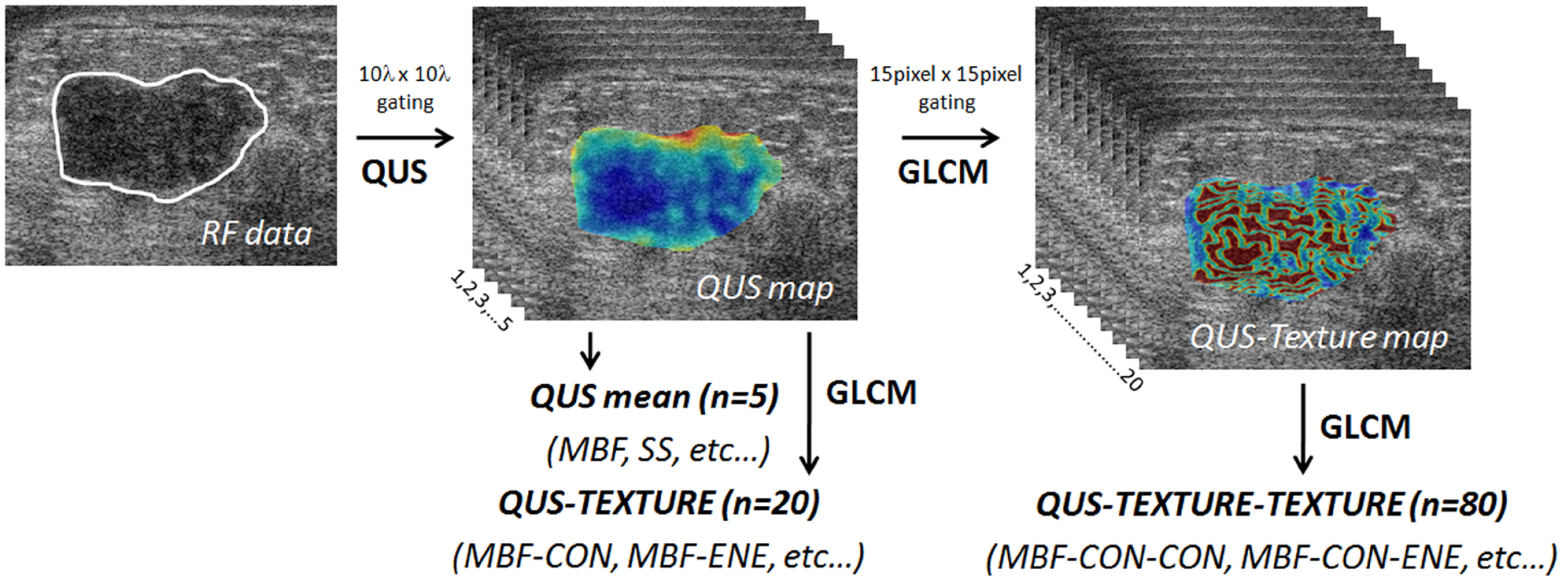
Generation of parametric and texture maps from radiofrequency data. Diagram showing flowchart of the generation of QUS parametric maps, texture, and texture derivative from radiofrequency ultrasound data. QUS: quantitative ultrasound; GLCM: grey level co-occurrence matrix.

For individual QUS features, mean values were determined by averaging QUS values obtained from all the sub-volumes. Four texture features, including CON, ENE, COR, and HOM, were evaluated using a GLCM method to generate QUS-Tex^1^ features from the QUS parametric maps. Corresponding color-coded QUS-texture feature maps for each QUS parametric map were constructed by making a spatial map of the texture values computed over all the sub-ROI. Subsequently, a second-pass texture analysis using the QUS parametric maps as input was applied to create QUS-Tex^1^-Tex^2^ features (texture-of-texture features) (Figure 4). A total number of 105 features (5 QUS, 20 QUS-Tex^1^, 80 QUS-Tex^1^-Tex^2^) were determined from ultrasound data for each tumor.

### Statistical analysis and classification algorithms

The mean values for all features (QUS, QUS-Tex^1^, QUS-Tex^1^-Tex^2^) between the two response groups were compared. A Shapiro-Wilk normality test was undertaken on feature-based data to determine its distribution. An unpaired *t*-test was completed for normally distributed data; otherwise, a Mann-Whitney test was used. The distribution of categorical variables between the two response groups was studied using the Pearson chi-square test and Fisher’s exact test as indicated. The continuous variables (age, tumor size) were compared using an unpaired *t*-test or the Mann-Whitney test as appropriate. A value of *p* < 0.05 was considered to be statistically significant. In this study, three multi-feature machine learning classifiers were used to classify patients into the two response groups. These included FLD, KNN, and SVM analyses. Classification analyses were performed using two data sets independently; (i) QUS means, and QUS-Tex^1^; or (ii) QUS means, QUS-Tex^1^ and QUS-Tex^1^-Tex^2^. Receiver operating characteristics (ROC) were determined for each classifier-based algorithm (responders versus non-responders) to obtain the AUC, and other indices like sensitivity, specificity, and accuracy were also calculated. Tumor segmentation, QUS data extraction, and classification analyses were performed using MATLAB R2016B (Mathworks, USA). Before performing class analyses, the class imbalance problem was circumvented by subsampling the original data into seven subsets, such that each unit had an equal number of responders and non-responder selected randomly. The feature selection was performed based on a sequential-forward selection method. Analyses permitted a maximum of 3 features in the classification model to avoid overfitting of the model given the number of samples in the cohorts used. Leave one out cross-validation was adopted to test the reliability of the classifiers. Survival analysis was done using a Kaplan Meier product-limit method with the factors compared using a log-rank test.

## CONCLUSIONS

In summary, the work here reports ultrasound radiomics can detect tumor response before neoadjuvant chemotherapy with high accuracy using texture derivative analysis of parametric images using a machine learning approach. The use of quantitative ultrasound-texture derivative biomarkers can serve as pretreatment survival-linked surrogates of response to cancer-targeting therapies leading the way towards personalized medicine and facilitate in selecting appropriate treatment regimen on an individual patient basis.

## SUPPLEMENTARY MATERIALS





## Abbreviations

QUS: Quantitative ultrasound
NAC: Neoadjuvant chemotherapy
LABC: Locally advanced breast cancer
QUS-Tex^1^: Quantitative ultrasound-texture
QUS-Tex^1^-Tex^2^: Quantitative ultrasound-texture derivatives
FLD: Fisher’s Linear discriminant
KNN: *k*-nearest neighbor
AUC: Area under curve
RFS: Recurrence-free survival
USG: Ultrasonography
MMG: Mammography
MRI: Magnetic resonance imaging
CT: Computed tomography
PET: Positron emission tomography
RF: Radiofrequency
ASD: Average scatterer diameter
AAC: Average acoustic concentration
MBF: Mid-band fit
SS: Spectral slope
SI: Spectral 0-MHz intercept
GLCM: Grey-level-co-occurrence matrix
HER 2: Human epidermal growth factor 2
R: Responder
NR: Non-responder
ROI: Region of interest
CON: Contrast
ENE: Energy
COR: Correlation
HOM: Homogeneity
ROC: Receiver operating characteristics
AC-T: Adriamycin, Cyclophosphamide, Paclitaxel
FEC-D: 5-Fluorouracil, Epirubicin, Cyclophosphamide, Docetaxel
pCR: Pathological complete response
