# Supplementary material for: Quantitative ultrasound radiomics using texture derivatives in prediction of treatment response to neo-adjuvant chemotherapy for locally advanced breast cancer
Source: Oncotarget. 2020 Oct 20;11(42):3782–92. doi: 10.18632/oncotarget.27742 (PMC7584238; doi:10.18632/oncotarget.27742)
Supplement: Supplementary file 1 [file oncotarget-11-3782-s001.pdf]

Quantitative ultrasound radiomics using texture derivatives in prediction of treatment response to neo-adjuvant chemotherapy for locally advanced breast cancer

SUPPLEMENTARY MATERIALS

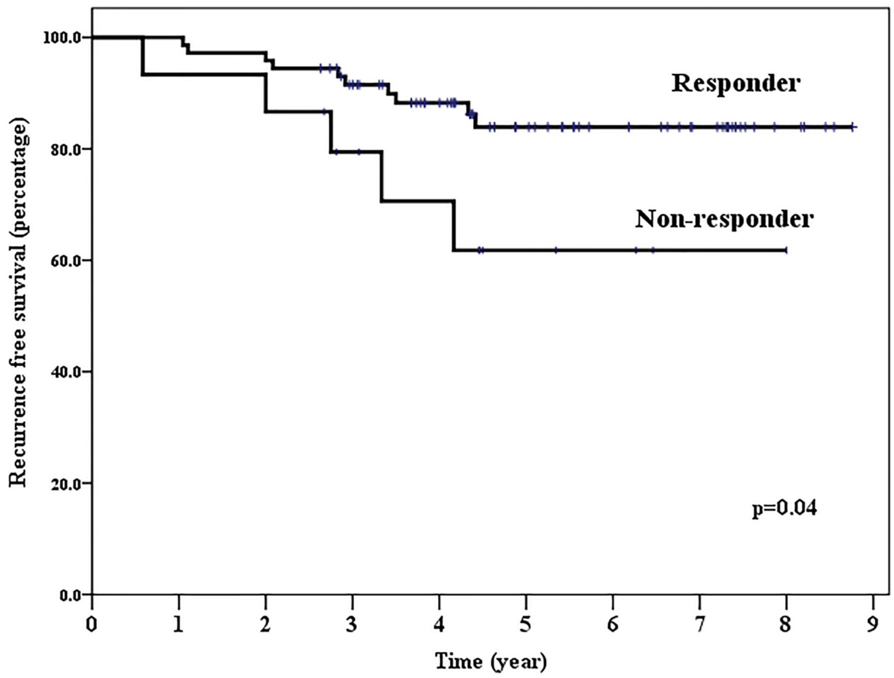

Supplementary Figure 1: Recurrence-free survival according to the treatment response. Kaplan Meier survival plot showing recurrence-free survival for the responders and non-responders.

Supplementary Table 1: Clinical information and treatment details for individual patients included in the study. See Supplementary Table 1
