# Supplementary material for: Quantitative ultrasound radiomics using texture derivatives in prediction of treatment response to neo-adjuvant chemotherapy for locally advanced breast cancer
Source: Oncotarget. 2020 Oct 20;11(42):3782–92. doi: 10.18632/oncotarget.27742 (PMC7584238; doi:10.18632/oncotarget.27742)
Supplement: Supplementary file 2 [file oncotarget-11-3782-s002.docx]

**Supplementary Table 1: Clinical information and treatment details for individual patients included in the study**

| **Serial no.** | **Age** | **Pre-treatment tumour size^a^**  **(cm)** | **ER** | **PR** | **HER2** | **T stage** | **N stage** | **Treatment** | **Trastuzumab** | **Post-treatment tumour size^b^**  **(cm)** | **Response** |
| --- | --- | --- | --- | --- | --- | --- | --- | --- | --- | --- | --- |
| 1 | 55 | 5.40 | - | - | + | T4 | N0 | FEC-D | Yes | 0.00 | R |
| 2 | 53 | 7.30 | + | + | - | T3 | N2 | ED | No | 7.00 | R |
| 3 | 43 | 5.30 | + | + | + | T3 | N1 | TC | Yes | 2.70 | NR |
| 4 | 67 | 10.00 | - | - | - | T4 | N1 | AC-D | No | 1.60 | R |
| 5 | 50 | 4.60 | + | + | + | T2 | N1 | AC-D | Yes | 0.00 | R |
| 6 | 33 | 5.00 | + | + | - | T2 | N0 | AC-T | No | 1.40 | R |
| 7 | 33 | 8.00 | + | + | + | T3 | N1 | AC-T | Yes | 0.00 | R |
| 8 | 48 | 4.90 | + | + | - | T4 | N1 | AC-D | No | 1.40 | R |
| 9 | 36 | 5.80 | + | + | - | T3 | N3 | AC-D | No | 11.40 | NR |
| 10 | 40 | 4.40 | - | - | - | T2 | N0 | AC-T | No | 2.00 | R |
| 11 | 59 | 6.00 | - | - | - | T3 | N0 | AC-T | No | 2.60 | R |
| 12 | 38 | 9.20 | + | + | - | T3 | N1 | AC-T | No | 4.50 | R |
| 13 | 56 | 11.70 | - | - | - | T4 | N1 | AC-T | No | 9.40 | NR |
| 14 | 48 | 4.30 | + | + | + | T3 | N2 | AC-T | Yes | 5.00 | R |
| 15 | 50 | 5.00 | - | - | - | T2 | N2 | AC-T | No | 4.00 | R |
| 16 | 49 | 12.00 | - | - | + | T3 | N2 | AC-T | Yes | 2.00 | R |
| 17 | 47 | 7.00 | - | - | - | T3 | N2 | AC-T | No | 0.00 | R |
| 18 | 40 | 3.00 | - | + | + | T2 | N1 | AC-T | Yes | 0.00 | R |
| 19 | 56 | 3.20 | - | + | + | T2 | N1 | AC-T | Yes | 0.20 | R |
| 20 | 50 | 5.60 | - | - | + | T3 | N1 | AC-T | Yes | 0.10 | R |
| 21 | 47 | 5.20 | + | + | - | T3 | N1 | FEC-D | No | 6.50 | R |
| 22 | 52 | 4.10 | + | + | - | T2 | N1 | AC-T | No | 0.00 | R |
| 23 | 44 | 9.90 | + | + | + | T3 | N1 | AC-T | Yes | 2.00 | R |
| 24 | 38 | 9.00 | + | + | - | T4 | N2 | AC-T | No | 2.00 | R |
| 25 | 58 | 2.00 | - | - | - | T3 | N0 | AC-T | No | 0.20 | R |
| 26 | 35 | 5.90 | - | - | - | T3 | N1 | AC-T | No | 0.00 | R |
| 27 | 38 | 2.60 | - | - | + | T2 | N0 | AC-T | Yes | 0.00 | R |
| 28 | 47 | 9.90 | + | + | - | T4 | N0 | AC-T | No | 8.00 | R |
| 29 | 57 | 5.50 | - | - | - | T3 | N1 | AC-T | No | 0.00 | R |
| 30 | 47 | 7.40 | - | - | + | T3 | N1 | AC-T | Yes | 0.00 | R |
| 31 | 55 | 12.80 | + | + | - | T3 | N1 | AC-T | No | 17.00 | NR |
| 32 | 33 | 7.00 | + | + | + | T3 | N2 | AC-T | Yes | 7.40 | NR |
| 33 | 38 | 2.50 | - | - | - | T2 | N0 | AC-T | No | 3.80 | NR |
| 34 | 55 | 10.50 | - | - | - | T3 | N1 | AC-T | No | 0.10 | R |
| 35 | 60 | 8.00 | + | - | + | T3 | N1 | FEC-D | Yes | 0.00 | R |
| 36 | 37 | 3.60 | + | + | - | T2 | N1 | AC-T | No | 2.20 | R |
| 37 | 49 | 9.00 | + | + | + | T4 | N1 | AC-T | Yes | 1.20 | R |
| 38 | 54 | 3.60 | + | + | - | T2 | N1 | TC | No | 1.70 | R |
| 39 | 55 | 1.60 | + | - | - | T2 | N1 | FEC-D | No | 1.20 | R |
| 40 | 50 | 7.30 | + | - | - | T3 | N1 | FEC-D | No | 2.10 | R |
| 41 | 55 | 3.40 | - | - | - | T2 | N0 | TC | No | 1.80 | R |
| 42 | 44 | 3.50 | - | - | - | T2 | N1 | FEC-D | No | 0.00 | R |
| 43 | 60 | 9.00 | + | - | - | T3 | N1 | FEC-D | No | 8.00 | R |
| 44 | 64 | 8.70 | + | + | - | T4 | N3 | FEC-D | No | 19.00 | NR |
| 45 | 66 | 2.50 | - | - | - | T2 | N0 | FEC-D | No | 3.20 | NR |
| 46 | 52 | 2.60 | - | - | - | T2 | N0 | FEC-D | No | 2.40 | R |
| 47 | 56 | 7.00 | + | + | + | T3 | N2 | AC-T | Yes | 8.40 | R |
| 48 | 45 | 2.30 | + | + | + | T4 | N1 | FEC-D | Yes | 0.00 | R |
| 49 | 59 | 4.90 | + | + | - | T2 | N0 | FEC-D | No | 2.80 | NR |
| 50 | 66 | 5.20 | + | + | + | T3 | N1 | TC | Yes | 4.00 | R |
| 51 | 49 | 2.10 | + | + | + | T2 | N0 | AC-T | Yes | 1.60 | R |
| 52 | 39 | 6.30 | + | + | - | T4 | N1 | FEC-D | No | 1.70 | R |
| 53 | 31 | 6.30 | - | - | - | T3 | N0 | AC-T | No | 12.60 | NR |
| 54 | 58 | 5.20 | + | + | + | T3 | N0 | AC-T | Yes | 3.40 | R |
| 55 | 51 | 4.00 | - | - | + | T2 | N1 | TC | Yes | 0.00 | R |
| 56 | 29 | 4.20 | + | + | - | T2 | N1 | AC-T | No | 4.00 | NR |
| 57 | 80 | 3.90 | + | - | + | T2 | N1 | AC-T | Yes | <1 | R |
| 58 | 43 | 9.60 | + | + | - | T3 | N1 | FEC-D | No | 3.00 | R |
| 59 | 66 | 3.00 | + | - | + | T2 | N1 | FEC-D | Yes | 2.40 | NR |
| 60 | 39 | 5.00 | - | - | - | T2 | N1 | AC-T | No | 5.00 | NR |
| 61 | 41 | 11.70 | + | + | + | T3 | N2 | AC-T | Yes | 1.30 | R |
| 62 | 54 | 8.80 | - | - | - | T4 | N1 | FEC-D | No | 2.50 | R |
| 63 | 48 | 3.50 | + | + | - | T2 | N0 | AC-T | No | 4.00 | R |
| 64 | 58 | 3.90 | + | - | - | T2 | N0 | AC-T | No | 3.30 | R |
| 65 | 64 | 3.00 | + | + | - | T2 | N0 | FEC-D | No | 0.50 | R |
| 66 | 48 | 2.40 | - | - | - | T2 | N3 | AC-T | No | 0.00 | R |
| 67 | 41 | 7.90 | + | + | + | T3 | N1 | AC-T | Yes | 0.20 | R |
| 68 | 43 | 6.60 | + | + | - | T3 | N0 | AC-T | No | 4.00 | R |
| 69 | 39 | 4.80 | - | - | - | T2 | N1 | AC | No | 0.00 | R |
| 70 | 70 | 4.30 | + | + | - | T2 | N1 | FEC-D | No | 2.00 | R |
| 71 | 52 | 4.20 | - | - | + | T2 | N1 | AC-T | Yes | 0.00 | R |
| 72 | 54 | 5.60 | - | - | + | T3 | N1 | AC-T | Yes | 0.20 | R |
| 73 | 55 | 7.90 | + | + | - | T3 | N1 | AC-T | No | 5.60 | R |
| 74 | 51 | 2.20 | + | + | - | T2 | N1 | AC-T | No | 0.50 | R |
| 75 | 56 | 3.10 | + | - | + | T2 | N1 | FEC-D | Yes | 0.00 | R |
| 76 | 32 | 4.00 | + | + | - | T2 | N1 | AC-T | No | 1.70 | R |
| 77 | 42 | 2.20 | + | + | - | T2 | N1 | AC-T | No | 1.10 | R |
| 78 | 53 | 2.30 | + | + | - | T3 | N0 | FEC-D | No | 0.00 | R |
| 79 | 42 | 3.10 | - | - | - | T2 | N1 | AC-T | No | 0.00 | R |
| 80 | 31 | 3.50 | - | - | - | T3 | N1 | AC-T | No | 0.00 | R |
| 81 | 33 | 5.60 | - | - | - | T3 | N1 | AC-T | No | 2.70 | R |
| 82 | 46 | 8.10 | - | - | + | T3 | N0 | TC | Yes | 1.60 | R |
| 83 | 53 | 5.30 | - | - | - | T3 | N1 | FEC-D | No | 0.00 | R |
| 84 | 59 | 5.30 | + | + | + | T3 | N1 | FEC-D | Yes | 1.50 | R |
| 85 | 38 | 10.90 | + | + | - | T3 | N0 | FEC-D | No | 4.90 | NR |
| 86 | 73 | 3.30 | + | - | - | T2 | N0 | AC-T | No | 0.20 | R |
| 87 | 42 | 4.50 | - | - | - | T2 | N1 | AC-T | No | 2.00 | R |
| 88 | 48 | 5.60 | + | + | - | T3 | N0 | FEC-D | No | 5.00 | R |
| 89 | 84 | 7.70 | + | - | - | T3 | N1 | AC-T | No | 0.00 | R |
| 90 | 51 | 4.50 | + | + | - | T2 | N3 | AC-T | No | 3.50 | NR |
| 91 | 43 | 9.00 | - | - | - | T3 | N0 | AC-T | No | 3.90 | R |
| 92 | 42 | 5.00 | + | + | - | T3 | N1 | FEC-D | No | 8.00 | NR |
| 93 | 60 | 7.20 | + | + | + | T4 | N3 | AC-T | Yes | 1.00 | R |
| 94 | 42 | 6.30 | + | + | - | T2 | N1 | FEC-D | No | 8.40 | NR |
| 95 | 47 | 10.40 | + | + | - | T2 | N1 | AC-T | No | 4.50 | R |
| 96 | 45 | 7.30 | + | + | - | T2 | N1 | FEC-D | No | 1.4 | R |
| 97 | 44 | 8.50 | - | - | - | T3 | N1 | FEC-D | No | <0.01 | R |
| 98 | 63 | 8.60 | + | + | - | T3 | N1 | AC-T | No | 7.5 | R |
| 99 | 52 | 3.10 | - | - | - | T2 | N0 | FEC-D | No | 0.2 | R |
| 100 | 61 | 8.20 | + | + | - | T3 | N3 | AC-T | No | 3.0 | R |

Abbreviations: ER+/PR+: estrogen/progesterone-receptor positive status; HER2+: human epidermal growth factor receptor 2 positive status; FEC-D: 5-fluorouracil, epirubicin, cyclophosphamide, and docetaxel; ED: Epirubicin, and Docetaxel; AC-T: Doxorubicin (Adriamycin) and cyclophosphamide followed by Paclitaxel; AC-D: Doxorubicin (Adriamycin) and cyclophosphamide followed by Docetaxel; TC: Platinum and Taxane; T: Taxane alone, no anthracycline; R: Responder; NR: Non-responder.

^a^ Maximum tumor size measured clinically and radiologically before initiation of any treatment.

^b^ Maximum dimension of the viable focus of tumor or span of the tumor bed (when multiple foci of residual tumor) from the pathological specimen.
